# Is Butyrate Concentration in the Equine Gastrointestinal Tract Altered During and After Surgery for Treatment of Large Colon Obstruction?

**DOI:** 10.3390/ani14223203

**Published:** 2024-11-08

**Authors:** Charlotte K. Barton, Diana M. Hassel, Kelly Anders, Tiffany L. Weir

**Affiliations:** Department of Clinical Sciences, College of Veterinary Medicine and Biomedical Sciences, Colorado State University, Fort Collins, CO 80523, USAtiffany.weir@colostate.edu (T.L.W.)

**Keywords:** butyrate, colon, colic, mucosa, volvulus

## Abstract

A major cause of morbidity and mortality in horses with large colon obstructive lesions is injury to the colonic mucosal barrier from ischemic injury. Since butyrate has been shown to play a critical role in the maintenance of a healthy mucosal barrier, it may play a role in the recovery process. This study’s objective was to determine whether the differences in butyrate concentrations existed between horses with surgical large colon obstructive lesions and healthy horses both during and after surgery. Eleven horses presenting with surgical colic lesions were enrolled; colonic samples were acquired during surgery, and fecal samples were obtained 36 h later. Colonic and fecal samples were also obtained from control groups. Samples were analyzed for butyrate, acetate, and propionate concentrations. There was no significant difference in butyrate content between surgical colonic or fecal samples and controls; however, an alteration in the proportion of SCFAs in relation to one another was noted. These changes in the individual SCFA levels were not statistically significant. The study findings demonstrated that there were no significant differences in butyrate proportions when comparing samples from horses with surgical colic lesions to healthy control horses.

## Simple Summary

Short-chain fatty acids have been shown to play a role both in the maintenance of a healthy mucosal barrier and the recovery following injury. The objective of this study is to determine whether there are differences in butyrate concentrations between horses with surgical large colon obstructive lesions and healthy horses both during and after surgery. Three key short-chain fatty acids were assessed: butyrate; propionate; and acetate. We did not find any significant differences between butyrate content in the colon of horses undergoing surgical intervention for large colonic obstructive lesions and healthy controls; additionally, there was no difference in fecal butyrate content and controls within the initial post-operative period. Despite this, we noted an alteration in the proportions of the three short-chain fatty acids in relation to one another. While these differences were not statistically significant, they represented an area requiring further research to fully investigate the roles of short-chain fatty acids in equine large colon obstructive lesions.

## 1. Introduction

Colic is a major medical problem seen in horses around the world. One of the most common and detrimental forms of colic that occurs in horses is large colon obstruction. A major cause of morbidity and mortality in these cases is injury to the colonic mucosal barrier from ischemic injury. The production of short-chain fatty acids (SCFAs) in the intestinal lumen is important for normal intestinal biology and serves as a primary energy source in horses. The predominant SCFAs in the equine colon are acetic acid, butyric acid, and propionic acid, which are produced by the microbial hydrolysis of dietary fiber, releasing soluble sugars that are fermented into SCFAs. These SCFAs have been shown to help improve colon mucosal health by modulating growth and cell differentiation [1]. A previous in vitro model of the intestinal epithelial barrier demonstrated that SCFAs, especially butyrate, could enhance intestinal barrier function as assessed by increases in transepithelial electrical resistance [2]. It has been proposed that this enhancement in intestinal barrier function was mediated by the role of butyrate in the regulation of the tight junction proteins via the AMPK pathway [3,4]. Butyrate also constitutes the major energy source for colonocytes [5] and plays a key role in intestinal immunity [6].

In addition to the tight junctions between intestinal epithelial cells, further colonic mucosal protection is provided by a mucus gel that is mainly composed of the secreted mucin, MUC2 [7]. This mucus barrier is disrupted when the colon becomes inflamed, allowing for the adherence of bacteria to epithelial tissue [8]. The mucus layer is directly in contact with the SCFAs, and butyrate has been shown to induce a six-fold increase in MUC2 gene expression; however, it produced a concurrent decrease in the mucus gel thickness [7]. While SCFAs’ exact role in the regulation of the mucus layer is not fully understood, alterations in butyrate likely play a role in impaired protective function.

Since SCFAs have a critical role in the establishment and maintenance of a healthy intestinal mucosal barrier, they may play a role in aiding in the recovery process following mucosal damage. In humans with Crohn’s disease, the number of butyrate-producing bacteria is decreased, and this has been shown to result in metabolic stress of the colonocytes [9]. Butyrate significantly reduces bacterial translocation across damaged epithelium, so prebiotics as a substrate for butyrate synthesis are valuable prophylaxes used for the regulation of epithelial permeability [9]. In horses with colic involving the large intestine, changes in fecal microbiota have been reported [10], and the impact of these changes on SCFA concentrations has yet to be determined.

In addition to providing protection to the healthy colon, butyrate has been shown to have some positive effects on colonic healing. In rats, butyrate was successfully used to help prevent ischemic reperfusion injury in the colon [11]. Additionally, it has been shown that intraluminal infusion of a mixture of short-chain fatty acids helped to promote anastomotic healing of the colon [1]. If butyrate concentrations are found to be reduced in the colon of horses undergoing celiotomy for treatment of large colon obstruction, it is plausible to consider butyrate supplementation as a mechanism to improve colonic health and serve as a preventative therapeutic for colic or as a treatment modality in horses with colonic injury following surgical treatment of colic.

The objective of this study was to demonstrate whether differences in butyrate concentrations existed between horses affected by surgical forms of large colon obstruction and horses with no gastrointestinal disease both during and after surgery. We hypothesized that horses diagnosed with a large colon obstruction had decreased levels of butyrate both during and after surgery when compared with horses unaffected by gastrointestinal disease.

## 2. Materials and Methods

All procedures were approved by the Clinical Research Review Board in the Department of Clinical Sciences at Colorado State University (protocol 2367, approved 6 July 2021), and a signed client consent was obtained for all procedures that were approved by the Institutional Animal Care and Use Committee prior to initiation of the study protocol.

### 2.1. Animals and Experimental Design

This was a prospective cohort study design. Eleven adult horses presenting to the Colorado State University Johnson Family Equine Hospital with large colon obstructive lesions that required surgical intervention were enrolled. All cases required a pelvic flexure enterotomy as part of the surgical intervention due to the nature of their lesions, and this was not influenced by enrollment in this study. Twenty-two horses were recruited as controls. The first control group included 11 horses receiving perioperative antibiotics and undergoing general anesthesia for an elective procedure not involving the gastrointestinal system (post-op fecal control group). The second control group included 11 horses presenting for euthanasia due to reasons unrelated to the gastrointestinal tract that had not received antibiotic therapy prior to presentation (colonic content control group). Exclusion criteria included horses less than 1 year of age and surgical colic patients not undergoing pelvic flexure enterotomy as a component of surgical management of their condition.

Sample size calculations were based on data from human studies of butyrate in healthy controls versus those with colorectal cancer from the collaborating laboratory performing the assays for this study, as equine data were unavailable. A sample size of 11 horses per group (*n* = 33 horses) was determined using an anticipated true difference of 9% with a standard deviation of 7% in butyrate concentrations between controls and cases. Enrollment of 11 horses per group was selected as it allowed for the rejection of the null hypothesis that the case and control groups are equal, with a power of 0.8 and an alpha of 0.05 (PSver3.1.2).

### 2.2. Sample Collection

Two sample sets were obtained from horses undergoing surgical treatment for large colon obstructive lesions. The intra-operative colonic content sample was obtained directly from the colon during the pelvic flexure enterotomy, and the post-operative sample was obtained 36 h later from feces removed directly from the rectum. The post-operative control group had fecal material obtained directly from the rectum 36 h following recovery from anesthesia. The euthanasia group served as colonic content controls. Immediately following euthanasia, a pelvic flexure enterotomy was performed, and colonic content was harvested. Once the samples were collected, they were flash-frozen at −80 °C within five minutes of collection, followed by storage at −80 °C or −20 °C to preserve SCFA levels for testing. In order to optimize sample quality, a preliminary analysis of SCFAs was performed with duplicates of the first 3 colonic and fecal samples, and these were analyzed as whole samples or strained samples, removing fiber content to determine if fiber content negatively impacted SCFA concentrations. All subsequent samples underwent straining through a single layer of gauze to remove large fiber particles as straining resulted in increased quantities of butyrate compared to identical samples inclusive of large fiber particles. When extrusion of fluid from fecal samples was not possible due to desiccation, whole feces were submitted for analysis.

### 2.3. Analysis of Samples

SCFA concentration analyses followed the same method described by Weir and Manter et al. [12]. Briefly, fecal and colonic samples were extracted for SCFAs by mixing 1 g of frozen feces with acidified water, followed by sonication, centrifugation, and filtering. Metabolites were detected using a Trace GC ultra with a Thermo DSQ II (Thermo Fisher Scientific, Waltham, MA, USA). This scanned from *m*/*z* 50–300 at a rate of 5 scans/s in an electric impact mode, as described. The inlet was held at 22 °C, and the transfer line was held at 230 °C. Samples were entered at a 10:1 split ratio. The temperature program consisted of 100 °C for one minute and then increasing 8 °C every minute until 180 °C was reached. The temperature of 180 °C was then held for one minute, and then the temperature was increased again to 200 °C at 20 °C per minute and held for another 5 min. Helium carrier flow was held at 1.2 mL per minute. All samples were run in duplicate with mean value calculations being determined using Thermo Quan software (GC Chemstation C.01.05) for butyrate, propionate, and acetate.

### 2.4. Statistical Analysis

Results were converted from mmol/mg concentrations into the proportion of the individual SCFA concentration relative to the total SCFAs obtained from the individual sample. The data were assessed for normality using the Shapiro–Wilks test, which confirmed that the data were not normally distributed, and, therefore, non-parametric testing was utilized. Statistical differences between colonic and fecal samples from horses with large colon obstruction and hospital controls were determined using a Wilcoxon rank sum test. The test analyzed differences in butyrate content between horses with large colon obstruction compared to both colonic controls and post-operative elective surgery fecal controls. A similar procedure was performed for propionate and acetate concentration. For within-horse sample analyses (intra-op colonic sample and post-operative fecal sample from colonic obstruction cases), a Wilcoxon signed-rank test was used to compare these two paired time points in colic horses. A separate analysis using a similar methodology was performed, including 8 of the 11 paired samples from surgical controls due to a change in sample storage that occurred following sample collection from the first 3 surgical cases. Due to a loss of −80 °C storage space from a freezer malfunction, all subsequent samples were stored at −20 °C. To account for a possible discrepancy from changes in storage conditions, analyses were performed in duplicate with and without the 3 paired colic samples. Additionally, the analysis of samples from horses with strangulating large colon lesions was compared to samples from horses with non-strangulating obstructive lesions. Wilcoxon rank sum tests were used to compare butyrate between these two populations.

## 3. Results

### 3.1. Demographics and Sampling

Samples from 33 horses were included in the final analysis. The average age of horses was 10.9 years old (range 2–23). Sixteen horses were Quarter Horses (48%); five were Warmbloods (15%); four were Arabians (12%); three were Draft breeds (9%); two were Thoroughbreds (6%), and three were unknown breeds (9%). There were no differences between the groups in terms of horse signalment, with the average age being 11.1 years old in the colic group, 10.1 in the post-operative control group, and 11.4 in the colonic control group.

**Figure 1:**
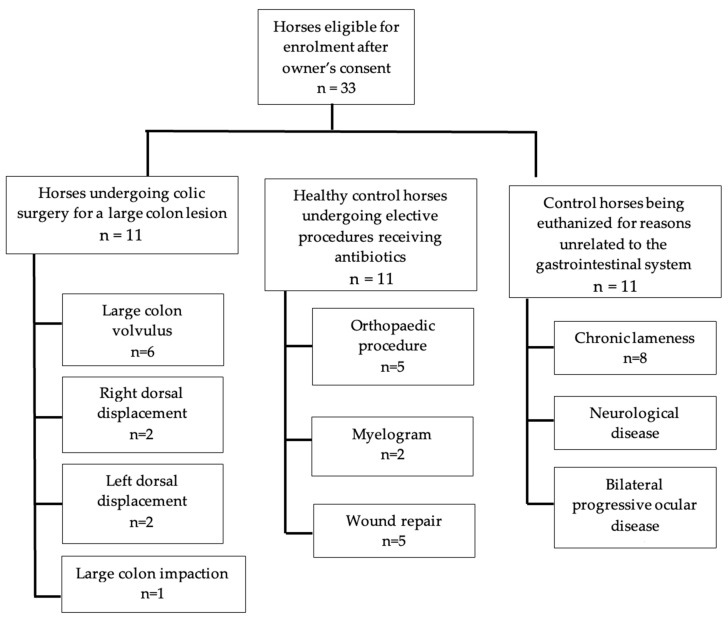
Flow chart of the horses enrolled in this study and the surgical procedures or lesions identified within each group.

Of the 11 horses presenting with signs of colic requiring surgical intervention, there were seven mares and four geldings. Breeds included Quarter Horses (*n =* 5, 45%), Warmbloods (*n =* 2, 18%), Draft Breeds (*n =* 2, 18%), Thoroughbred (*n =* 1, 9%), and an Arabian (*n =* 1, 9%). Five horses were diagnosed with a large colon volvulus at the time of surgery, two horses had left dorsal colonic displacements, two horses had right dorsal colonic displacements and one horse with a large colon impaction (Figure 1). The average time between the onset of colic signs and presentation at the hospital was less than 12 h, with three cases presenting 12–14 h later and one case presenting 24–36 h later. All horses received broad-spectrum antibiotics consisting of potassium–penicillin (22,000 IU/kg q6) and gentamicin (6.6 mg/kg IV q24), beginning immediately prior to anesthetic induction and continuing for a minimum of 24 h.

The control group presenting for elective procedures to be performed under general anesthesia and receiving perioperative antibiotics contained 11 horses. The group included three mares and eight geldings. Breeds represented were Quarter Horses (*n =* 4, 36%), Warmbloods (*n =* 2, 18%), Arabians (*n =* 2, 18%), a Draft Breed (*n =* 1, 9%), a Thoroughbred (*n =* 1, 9%), and an unknown breed (*n =* 1, 9%). Five horses underwent orthopedic procedures (45%); two horses received myelograms (18%), and four horses were anesthetized for wound repair (36%). Antibiotics were administered perioperatively for all control anesthetized horses, and these consisted of five horses (45%) receiving potassium–penicillin (22,000 IU/kg q6) and gentamicin (6.6 mg/kg IV q24), four horses (36%) receiving cefazolin alone (10 mg/kg IV q8hr), or cefazolin (10 mg/kg IV q8hr) and gentamicin (6.6 mg/kg IV q24) combined in two horses (18%).

The euthanized control (colonic sample) group also contained 11 horses consisting of Quarter Horses (*n =* 7, 63%), Arabians (*n =* 1, 9%), Warmbloods (*n =* 1, 9%), and unknown breeds (*n =* 2, 18%). Of the donated horses, eight were presented for chronic lameness (72%), two for neurological disease (18%), and one for chronic progressive bilateral ocular disease (9%).

### 3.2. Butyrate

The mean total proportions of butyrate in samples taken from horses presenting with surgical colic lesions were 5.88% in the colonic samples taken intra-operatively and 1.07% in the fecal samples obtained 36 h post-operatively. This decrease in butyrate was statistically significant (*p* = 0.003, Table 1). There were no statistically significant differences in butyrate proportion between horses presenting with strangulating lesions compared with non-strangulating obstructive lesions.

**Figure 2:**
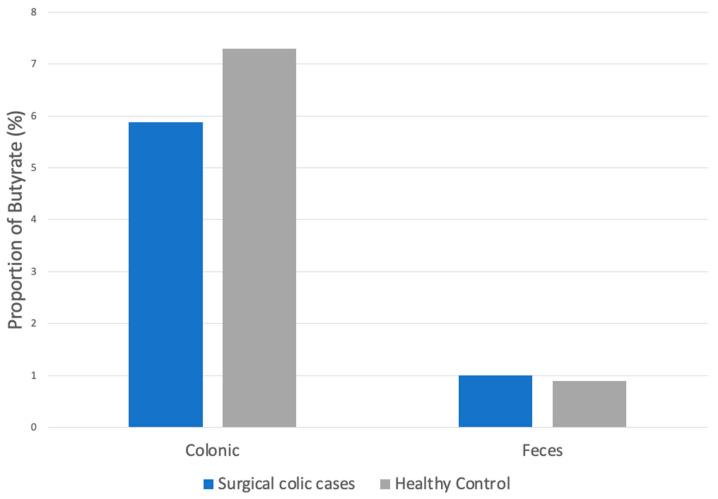
Proportion of butyrate in colonic and fecal samples of horses presenting for surgical colic compared to healthy controls.

The proportion of butyrate from the colon of horses with surgical colic lesions was 1.9% lower when compared to colonic butyrate in control cases; however, this decrease was not statistically significant (*p* = 0.3, Figure 2).

There was also no significant difference between the proportion of butyrate from the feces of horses with surgical lesions taken 36 h following surgery (1.07%) compared to butyrate from the feces of control horses (0.9%, *p* = 0.4). Three of 11 and 7 of 11 had butyrate concentrations below the limit of detection in the surgical post-operative fecal samples and control fecal samples, respectively. Therefore, the limit of detection for butyrate was used for statistical analysis. No significant changes in results for all three SCFAs were detected, with the exclusion of the initial three samples stored exclusively at −80 °C for all samples, comparing colic cases to control horses for both colonic and fecal analyses.

### 3.3. Propionate

**Figure 3:**
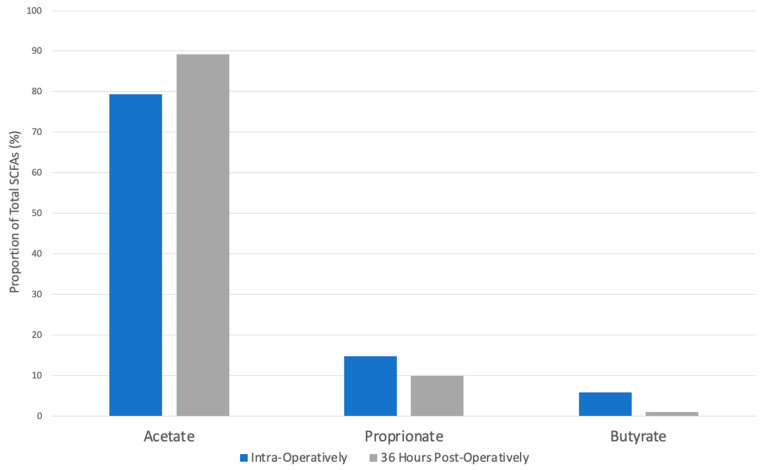
Proportions of SCFAs in horses presenting for surgical colic lesions in colonic samples obtained intra-operatively compared to fecal samples 36 h post-operatively. The star indicates a statistically significant difference (*p* < 0.05).

The total proportion of propionate in horses presenting with surgical colic lesions was 14.38% intra-operatively and 9.77% in fecal samples taken from the same horse 36 h later. This difference of 4.61% was not statistically significant (*p* = 0.06, Figure 3).

The proportion of propionate in horses with surgical colic lesions from samples obtained from the colon intra-operatively was 4.17% lower than colonic samples taken from healthy controls (18.55%). This was not statistically significant (*p* = 0.1).

Proprionate proportion was increased by 1.5% in fecal samples of horses with surgical lesions taken 36 h post-operatively (9.77%) compared to fecal samples obtained from healthy horses (8.27%). This difference was not statistically significant (*p* = 0.7).

### 3.4. Acetate

The total proportion of acetate in horses presenting with surgical colic lesions was 79.37% intra-operatively and 89.17% post-operatively. This difference of 9.22% was statistically significant (*p* = 0.006, Figure 3).

Acetate proportion in horses with surgical colic lesions in samples obtained from the colon at the time of surgery was 5.25% lower than colonic control samples from healthy horses (85.19%). This decrease was not statistically significant (*p* = 0.06).

The proportion of acetate in horses with surgical colic lesions obtained from fecal samples 36 h post-operatively was 15.75% higher compared to fecal samples from healthy control horses (73.42%). This increase was not statistically significant (*p* = 0.4).

## 4. Discussion

The findings of this study demonstrated that there was a significant reduction in the proportion of butyrate within fecal samples obtained 36 h post-operatively in horses undergoing surgery for treatment of colic due to large colon obstruction compared to intra-operative colonic samples obtained from a pelvic flexure enterotomy. In contrast, the proportion of acetate significantly increased. These differences in SCFA proportion were not observed when comparing colic intra-operative or post-operative samples to the two control populations that did not have gastrointestinal disease. Therefore, our hypothesis was rejected as SCFAs in both colonic and post-operative fecal samples were found to be similar to control horses without gastrointestinal dysfunction. While there were no significant differences in SCFA proportions between horses with large colon obstructive disease and controls, there was an overall alteration in the proportions of SCFAs in relation to one another. A trend toward lower butyrate, propionate, and acetate was observed in the colon of colic horses compared with controls (*p* = 0.3, *p* = 0.1, *p* = 0.06, respectively); yet, acetate and butyrate proportions trended higher in feces in the post-operative period of colics compared with control horse feces.

The role of a pelvic flexure enterotomy with intra-colonic water lavage and evacuation should be an important consideration when considering the reduction in the proportion of butyrate between intraoperative colonic samples and post-operative samples in horses with large colon obstructions. The proportions of SCFAs present within the colonic and rectal environment should reflect the types and activity of SCFA-producing bacteria. We know from prior research [10,13] that changes in the microbiota occur during the hospitalization of horses for treatment of colic. As SCFAs are a byproduct of bacterial fermentation of fiber, changes to the microbial environment are anticipated following colonic lavage along with the administration of systemic antimicrobial agents. Stewart et al. [13] recognized that horses presenting for colic had a significantly lower differential abundance of the phylum *Firmicutes*, specifically, reductions in the predominant commensal bacteria, *Clostridia* and *Lachnospiraceae*, known for their production of butyrate [14]. The reduction in the proportion of butyrate may also be reflective of normal differences that may exist between the ascending colon and rectum of horses, as these differences are not well-characterized. In addition, the large proportion of samples with butyrate levels below the limit of detection in both colic feces (*n* = 3/11) and control feces (*n* = 7/11) makes these differences likely erroneous. Desiccation of many of the fecal samples may have contributed to the assay’s limited ability to detect butyrate, as supported by our preliminary work showing higher butyrate content in colonic samples devoid of most fibrous material compared with identical raw samples containing fibrous material.

Observed changes in proportions of acetate and propionate between controls and colic cases also may be reflective of changes to the microbial populations or their activities as production of these SCFAs is dominated by differing bacterial communities than those that contribute to butyrate production. Brokner et al. demonstrated a beneficial metabolic response and higher caecal propionate concentrations in horses fed larger quantities of soluble fiber, suggestive of a healthier colonic environment [15]. Alternatively, these findings may represent spurious results as differences observed using proportions were less evident when analyzing direct concentrations of acetate and propionate.

In our control surgical population, some variation in antimicrobial choices may have had differing effects on the fecal microbial environment, but considering the short timeline from initial antimicrobial treatment to sampling (36 h) and potential delays in normal transit from general anesthesia, it is possible that the impact of any form of antimicrobial therapy may not yet be reflected in fecal samples.

The limitations of this study are largely due to the inherently volatile nature of the fatty acids making accurate extraction and quantification challenging. The first three samples were stored at −80 °C; however, due to a faulty refrigerator, all subsequent samples were flash-frozen at −80 °C to try and preserve VFA content but were then stored at −20 °C until testing was performed. Samples that were stored at −20 °C had less butyrate extracted from them compared to the samples stored at −80 °C, although acetate and propionate concentrations did not seem to be similarly reduced. This inconsistency was corrected within the results through a second analysis, excluding the three samples, but highlighted the difficulties in accurately preserving the VFAs. There were also multiple samples with butyrate below the limit of detection, and all samples where this occurred were derived from more desiccated fecal samples. Although it is anticipated that lower levels of SCFAs would be present in feces compared to colonic content, it is probable that the higher fiber present in the samples derived from the rectum may have falsely lowered the concentrations compared with colonic samples. Similarly, concentrations may have been impacted by fecal desiccation, leading to degradation of butyrate prior to freezing.

In this study, we elected to report results as proportions of butyrate, acetate, and propionate within each sample, as these three SCFAs represent the predominant volatile fatty acids (VFAs) found in the colonic and fecal environment. Other VFAs, such as valerate, isovalerate, and isobutyrate, were not evaluated in the present study as they collectively represent less than 10% of the total VFA in horse feces [16]. It is possible that these less predominant VFAs play a larger role in pathologic states, rendering the use of proportions of the three predominant SCFAs invalid. Raspa et al. demonstrated increases in valeric acid in horses fed a high starch diet compared to a high fiber diet [17]. By reporting proportions as the primary means of data analysis, rather than direct measurements of concentrations, we hoped to correct for potential discrepancies related to sample quality (e.g., desiccated feces vs. moist colonic content) as our preliminary analysis demonstrated more effective extraction of butyrate from the fiber-filtered samples.

While we ensured that our colonic control group did not receive antibiotics, the surgical colic horses undergoing colonic sampling received pre-operative antibiotics prior to induction. As these were administered 30 min prior to the time of the first incision, it is unlikely that they had any impact on the colonic microbiota due to the minimal time elapsed between administration and sampling. A further limitation of this study is the relatively low number of horses enrolled and the diversity of both horses and their diets. Diet has been demonstrated to influence the microbiota in horses [18,19,20,21] and, likely, would also affect SCFA concentrations.

Despite this study’s limitations, the results support further investigations into the role of butyrate and other SCFAs in equine intestinal health and repair. Future studies may include matching cases and controls by diet and environment and inclusion of additional VFAs along with microbiota analyses to further elucidate observed changes in SCFAs. The observation of a reduction in butyrate concentration in horses undergoing large colon enterotomy for the treatment of colic supports consideration of future research looking at intraluminal supplementation of butyrate to assess its impact on post-operative butyrate concentrations and health outcomes.

## 5. Conclusions

This study did not identify a statistically significant reduction in butyrate proportion in horses presenting with large colon obstructive lesions compared to healthy controls. However, alterations in the proportions of common SCFAs were observed, highlighting the need for further investigations into the role of SCFAs both in normal gastrointestinal health and recovery from disease.

## Figures and Tables

**Table 1:** Table shows the mean and standard deviation for the proportions (%) of the short-chain fatty acids in the surgical colic samples and the control groups.

	Butyrate		Propionate		Acetate	
	Mean	Standard Deviation	Mean	Standard Deviation	Mean	Standard Deviation
Surgical Colic Intraoperative Colonic Sample	5.9	7.3	14.4	6.7	79.4	5.2
Surgical Colic Post-Operative Fecal Sample	1.1	1.9	9.8	12.2	89.2	11.1
Colonic Control	7.3	7.3	18.6	5.2	73.4	7.7
Feces Control	0.9	2.1	8.3	8.9	90.8	7.7

## Data Availability

The original contributions presented in the study are included in the article, further inquiries can be directed to the corresponding authors.
